# Disparity in neonatal abstinence syndrome by race/ethnicity, socioeconomic status, and geography, in neonates ≥ 35 weeks gestational age

**DOI:** 10.1371/journal.pone.0284040

**Published:** 2023-04-05

**Authors:** Keith A. Dookeran, Marina G. Feffer, Kyla M. Quigley, Phoebe E. Troller, Chariya A. Christmon, Janine Y. Khan

**Affiliations:** 1 Joseph J. Zilber School of Public Health, University of Wisconsin-Milwaukee, Milwaukee, Wisconsin, United States of America; 2 Division of Epidemiology and Biostatistics, School of Public Health, University of Illinois at Chicago, Illinois, United States of America; 3 The Cancer Foundation for Minority and Underserved Populations, Chicago, Illinois, United States of America; 4 Loyola University Chicago, Chicago, Illinois, United States of America; 5 Cook County Department of Public Health, Chicago, Illinois, United States of America; 6 Department of Pediatrics, Ann & Robert H. Lurie Children’s Hospital, Northwestern University, Chicago, Illinois, United States of America; Montclair State University, UNITED STATES

## Abstract

Neonatal abstinence syndrome (NAS) is associated with a range of adverse health outcomes, exorbitant health care costs, and race/ethnicity disparity. We examined key sociodemographic factors that may influence the national race/ethnicity disparity in the prevalence of NAS among Whites, Blacks and Hispanics. 2016 and 2019 cycles of cross-sectional data from HCUP-KID national all-payer pediatric inpatient-care database were used to estimate NAS prevalence (ICD-10CM code P96.1) in newborns ≥ 35 weeks gestational-age, excluding iatrogenic-cases (ICD-10CM code P96.2). Multivariable generalized-linear-models with predictive-margins were used to produce race/ethnicity-specific stratified-estimates for select sociodemographic factors, reported as risk-differences (RD) with 95% confidence-intervals (CI). Final models were adjusted for sex, payer-type, ecologic income-level, and hospital size, type, and region. The overall survey weighted-sample prevalence of NAS was 0.98% (i.e., 6282/638100) and did not differ over cycles. Blacks and Hispanics were significantly more likely than Whites to be in the lowest ecologic income quartile and on Medicaid. In fully-specified models, NAS prevalence among Whites was 1.45% (95% CI: 1.33, 1.57) higher than Blacks and 1.52% (95% CI: 1.39, 1.64) higher than Hispanics; and NAS among Blacks was 0.14% higher than Hispanics (95% CI: 0.03, 0.24). NAS prevalence was highest among Whites on Medicaid (RD: 3.79%; 95% CI: 3.55, 4.03) compared to Whites on private-insurance (RD: 0.33%; 95% CI: 0.27, 0.38), and Blacks (RD: 0.73%; 95% CI: 0.63, 0.83; RD: 0.15%; 95% CI: 0.08, 0.21), or Hispanics, with either payer-type (RD: 0.59%; 95% CI: 0.5, 0.67; RD: 0.09%; 95% CI: 0.03, 0.15) respectively. NAS prevalence was higher among Whites in the lowest income-quartile (RD: 2.22%; 95% CI: 1.99, 2.44) compared with Blacks (RD: 0.51%; 95% CI: 0.41, 0.61) and Hispanics (RD: 0.44%; 95% CI: 0.33, 0.54) in the same quartile, and all subgroups in other quartiles. NAS prevalence was higher among Whites in the Northeast (RD: 2.19%; 95% CI: 1.89, 2.5) compared to Blacks (RD: 0.54%; 95% CI: 0.33, 0.74) and Hispanics (RD: 0.31%; 95% CI: 0.17, 0.45). Although Blacks and Hispanics were more likely to be in the lowest income quartile and have Medicaid insurance, Whites on Medicaid, in the lowest income quartile, and in the Northeast, were found to have the highest NAS prevalence.

## Introduction

Opioid use among women of reproductive age remains a persistent public health concern because of its association with adverse neonatal outcomes, including birth defects and neonatal abstinence syndrome (NAS) [1–3]. NAS is a postnatal drug withdrawal syndrome that occurs among opioid-exposed newborns shortly after birth, often manifested by central nervous system irritability, autonomic over-reactivity, and gastrointestinal dysfunction [4,5]. The prevalence of NAS has increased throughout the U.S. in the past two decades [6–12] and NAS is thought to be responsible for a substantial and growing portion of resources dedicated to neonates in neonatal intensive care units (NICUs) nationwide [10–14].

It has been previously reported that the national prevalence of NAS was found to be highest among non-Hispanic White (White) newborns [15], and 2015 Agency for Healthcare Research and Quality (AHRQ) data demonstrates NAS rates by race/ethnicity as 10.1 per/1000 births for Whites, 2.9/1000 for non-Hispanic Blacks (Blacks), and 2.3/1000 for Hispanics [15]. These reports further suggest that NAS may be more prevalent in rural settings, among lower-income families, and families enrolled in public insurance plans like Medicaid [8].

In addition, several states report high levels of NAS with sociodemographic features similar to those observed in national studies. For example, prior studies from Nevada and Louisiana report that NAS was disproportionately higher among White newborns and Medicaid enrollees [16,17]. Eaves et al. also reported that White and economically disadvantaged neonates of unmarried mothers in Arizona were more likely to be affected by opioid withdrawal [18]. Haward et al. reported that the Appalachian Mountain region in North Carolina, which has been widely associated with health disparities, had a NAS incidence rate nearly three times higher than the statewide rate [19].

The objective of our study is to examine key sociodemographic factors that may influence the national race/ethnicity disparity in the prevalence of NAS among Whites, Blacks and Hispanics. Some critically ill infants often require extended use of opioid medications for sedation that may result in iatrogenic withdrawal symptoms in newborns not exposed to opioids in the antenatal period [6], and it is therefore necessary to identify and exclude such cases when performing population-based studies on non-iatrogenic NAS. The most recent update (2019 cycle) of Kids’ Inpatient Database (KID) offers an opportunity to study recent NAS prevalence trends and to examine the relationship between key sociodemographic factors and U.S. national disparities in NAS.

## Study data and methods

We utilized recent data from the (Agency for Healthcare Research and Quality) AHRQ, Healthcare Cost and Utilization Project (HCUP) KID database. The KID is the largest publicly-available all-payer pediatric inpatient care database in the US, yielding national estimates of hospital inpatient stays by children (including newborns) and has been produced almost every three years since 1997 [20]. The KID target universe includes pediatric discharges from community, non-rehabilitation hospitals in the US. Inpatient stay records in the KID include clinical and resource use information typically available from discharge abstracts created by hospitals for billing, and it contains charge information on all patients, regardless of payer, including persons covered by private insurance, Medicaid, Medicare, and the uninsured [20].

The KID’s large sample size enables analyses of rare conditions, and it can be used to identify, track, and analyze national trends in healthcare utilization, cost, quality, and outcomes [20]. The number of States in the KID has grown from 22 in the first year (1997) to 48 plus the District of Columbia in 2019 [20]. Beginning with 2016 data, the KID includes the International Classification of Diseases, Tenth Revision (ICD-10) Clinical Modification (CM)/ Procedure Coding System (PCS) data only, and per AHRQ guidance this study utilized KID database statistical discharge weights to obtain national estimates [21]. We used available data from the 2016 and 2019 cycles, which when weighted, estimates roughly 7 million hospitalizations per year and the sample size was fixed. Both 2016 and 2019 KID database cycles include a sample of 10% of normal newborns and 80% of other pediatric discharges from more than 4,000 U.S. community hospitals [21].

Our study included newborns ≥ 35 weeks gestational age (GA) with a NAS diagnosis corresponding to ICD-10-CM diagnosis code P96.1 (i.e., neonatal withdrawal symptoms from maternal use of drugs of addiction) in any one of 30 KID discharge diagnostic fields. We specifically excluded iatrogenic NAS cases using ICD-10-CM diagnosis code P96.2 (i.e., withdrawal symptoms from therapeutic use of drugs in newborn). We also used the KID variable for in-hospital birth (I10_HOSPBIRTH) to identify in-hospital births, defined as those with principal or secondary diagnosis code indicating a live birth (i.e., ICD-10-CM codes Z38.00, Z38.01, Z38.2, Z38.30, Z38.31, Z38.5, Z38.61-Z38.69, Z38.8) and no indication of birth outside the hospital or transfer from another hospital [22].

In consultation with experienced clinicians involved in the treatment of newborns diagnosed with NAS (CAC and JYK), we performed a contextually informed systematic review of the KID data dictionary. All covariates related to NAS diagnosis and sociodemographic factors [22], were included in our analyses: sex (female/male); payer type (Medicaid/private); race/ethnicity (Black/White/Hispanic); quartile classification of the estimated median household income of residents in the patient’s ZIP Code as a measure of ecologic socioeconomic status (SES); hospital size (large/medium/small); hospital type by teaching status (rural/urban non-teaching/urban teaching); hospital geographic region (Northeast/Midwest/South/West); and KID cycle year (2016/2019).

We examined associations between selected sociodemographic factors and NAS status, and for categorical variables the Chi-Square Test of Association was used to examine the significance of relationships. Multivariable generalized linear regression models (GLM) with predictive margins [23–26] were used to estimate associations between binary race/ethnicity exposure models (i.e., White/Black, White/Hispanic, and Black/Hispanic) and NAS status, and are reported as risk differences (RDs) with 95% confidence intervals (CIs). Analytic covariates were examined for missingness and were found to have < 1% of missing data. Due to the small amount of missingness, only complete cases were used for analysis. Interaction terms between race/ethnicity and all other covariates were included in fully specified multivariable models to facilitate production of stratified-estimates, and final models for predicted mean risk of NAS were adjusted for sex, race, payer type, SES, and hospital size, type, and region. All analyses were conducted using Stata statistical software, version 17 (Stata Corp., College Station, TX), accounted for complex survey design, and utilized a common primary sampling unit survey weight variable across KID database cycles. All tests are two-sided with a threshold for significance of p<0.05. Our study used deidentified admission claims data from a public registry for secondary data analysis, and all HCUP-KID data are fully anonymized before data access is allowed, and as such, there is no requirement for informed consent. Before commencing our study, the University of Wisconsin-Milwaukee Institutional Review Board (UWM IRB) reviewed our research study protocol and determined that it did not meet the definition of human subject research, and as such, IRB review and approval was not required. In addition, our study methodology comports with all Strengthening the Reporting of Observational Studies in Epidemiology (STROBE) guidelines [27].

## Study results

Our survey weighted sample was representative of 638,100 newborn hospital discharges from 2016 and 2019. The overall weighted sample prevalence of NAS was 0.98% (i.e., 6282/638100) and did not differ over cycles (i.e., 2016: 1.02%; 2019: 0.95%). The distribution of select population characteristics by race/ethnicity is provided in Table 1. Compared to Whites, Blacks and Hispanics appeared significantly more likely to have Medicaid insurance (36% vs. 74% and 68%, respectively) and lower SES (i.e., lowest quartile distribution: 20% vs. 48% and 35%, respectively). The distribution by race/ethnicity was also similar over both database cycles (i.e., 2016/2019—Whites 50%/50%; Blacks: 47%/53%; Hispanics: 48%/52%).

**Table 1:** Distribution of select population characteristics by race/ethnicity.

		Total		White		Black		Hispanic		P-value**
Factor		N	(%*)	n	(%*)	n	(%*)	n	(%*)	
		580456		343818		102705		133933		
**Sex**										0.0478
** **	Male	282691	(0.52)	166931	(0.52)	49739	(0.51)	66020	(0.52)	
** **	Female	262328	(0.48)	154535	(0.48)	47374	(0.49)	60419	(0.48)	
**Hospital bed size**										<0.001
** **	Small	83705	(0.15)	53469	(0.16)	13492	(0.14)	16743	(0.13)	
** **	Medium	143747	(0.26)	87070	(0.27)	26790	(0.27)	29887	(0.23)	
** **	Large	317786	(0.59)	181064	(0.57)	56867	(0.59)	79855	(0.63)	
**Hospital location/teaching status**										<0.001
** **	Rural	38530	(0.07)	30409	(0.09)	4389	(0.04)	3732	(0.03)	
** **	Urban non-teaching	104840	(0.19)	67591	(0.20)	12887	(0.13)	24362	(0.19)	
** **	Urban teaching	401867	(0.74)	223603	(0.71)	79872	(0.83)	98392	(0.78)	
**Payer type**										<0.001
** **	Private	253895	(0.50)	191643	(0.64)	24086	(0.26)	38166	(0.32)	
** **	Medicaid	249971	(0.50)	105195	(0.36)	65591	(0.74)	79185	(0.68)	
**Ecologic median household income quartile** ***										<0.001
** **	Quartile 1 (highest)	117720	(0.21)	88140	(0.27)	10486	(0.11)	19094	(0.15)	
** **	Quartile 2	139638	(0.26)	90375	(0.28)	17886	(0.18)	31378	(0.25)	
** **	Quartile 3	131050	(0.24)	78146	(0.25)	22046	(0.23)	30858	(0.25)	
** **	Quartile 4 (lowest)	152264	(0.28)	62521	(0.20)	46025	(0.48)	43718	(0.35)	
**Year**										0.3429
** **	2016	269062	(0.49)	161219	(0.50)	46375	(0.47)	61468	(0.48)	
** **	2019	276176	(0.51)	160384	(0.50)	50774	(0.53)	65018	(0.52)	
**Hospital region**										<0.001
** **	Northeast	95837	(0.17)	62619	(0.19)	15971	(0.16)	17247	(0.13)	
** **	Midwest	97646	(0.18)	72316	(0.23)	16779	(0.18)	8551	(0.07)	
	South	208763	(0.38)	112933	(0.35)	54271	(0.55)	41559	(0.33)	
	West	142992	(0.26)	73735	(0.23)	10128	(0.10)	59129	(0.47)	

*Survey weighted percentages.

**Pearson Chi-Square Test.

***Based on patient’s ZIP Code.

The distribution of select population characteristics by NAS status is provided in Table 2. NAS was significantly more prevalent among Whites (14.72 per 1000 newborns vs. 6.02 and 4.3 per 1000 for Black and Hispanics respectively), those with Medicaid insurance payer type (18.54 per 1000 vs. 1.8 per 1000 for Private insurance), in rural hospitals (14.48 per 1000 vs. 8.14 and 9.87 per 1000 for urban non-teaching and urban teaching, respectively), in the Northeast and South hospital regions (11.38 and 11.58 per 1000 respectively, vs. 10.49 and 6.12 per 1000 for the Midwest and West respectively), and showed a monotonic increasing trend with decrease in ecologic median household income quartiles (all p < 0.05).

**Table 2:** Distribution of select population characteristics by NAS status.

		Total		Not NAS		NAS		P-value**	NAS Cases Per 1000 Population
		N	(%*)	n	(%*)	n	(%*)		
Factor									
		638100		631818		6282			9.84
**Sex**								0.51	
** **	Male	330899	(0.52)	327611	(0.52)	3288	(0.52)		9.94
** **	Female	306948	(0.48)	303958	(0.48)	2990	(0.48)		9.74
**Hospital bed size**								0.15	
** **	Small	95492	(0.15)	94666	(0.15)	826	(0.13)		8.65
** **	Medium	170068	(0.27)	168407	(0.27)	1661	(0.26)		9.77
** **	Large	372540	(0.58)	368745	(0.58)	3795	(0.60)		10.19
**Hospital location/teaching status**								<0.001	
** **	Rural	42366	(0.07)	41753	(0.07)	613	(0.10)		14.48
** **	Urban non-teaching	121127	(0.19)	120141	(0.19)	986	(0.16)		8.14
** **	Urban teaching	474607	(0.74)	469925	(0.74)	4682	(0.75)		9.87
**Race/ethnicity**								<0.001	
** **	White	321603	(0.59)	316868	(0.59)	4735	(0.81)		14.72
** **	Black	97149	(0.18)	96564	(0.18)	584	(0.10)		6.02
** **	Hispanic	126486	(0.23)	125942	(0.23)	544	(0.09)		4.30
**Payer type**								<0.001	
** **	Private	302074	(0.51)	301530	(0.52)	545	(0.09)		1.80
** **	Medicaid	287365	(0.49)	282039	(0.48)	5327	(0.91)		18.54
**Ecologic median household income quartile** ***								<0.001	
** **	Quartile 1 (highest)	150157	(0.24)	149412	(0.24)	745	(0.12)		4.96
** **	Quartile 2	162084	(0.26)	160708	(0.26)	1376	(0.22)		8.49
** **	Quartile 3	148578	(0.23)	146860	(0.23)	1718	(0.28)		11.57
** **	Quartile 4 (lowest)	171783	(0.27)	169429	(0.27)	2355	(0.38)		13.71
**Year**								0.23	
** **	2016	314522	(0.49)	311307	(0.49)	3215	(0.51)		10.22
** **	2019	323578	(0.51)	320511	(0.51)	3067	(0.49)		9.48
**Hospital region**								<0.001	
	Northeast	119206	(0.19)	117849	(0.19)	1357	(0.22)		11.38
	Midwest	107845	(0.17)	106714	(0.17)	1131	(0.18)		10.49
	South	234171	(0.37)	231460	(0.37)	2711	(0.43)		11.58
	West	176878	(0.28)	175794	(0.28)	1083	(0.17)		6.12

*Survey weighted percentages.

**Pearson Chi-Square Test.

***Based on patient’s ZIP Code.

**Fig 1:**
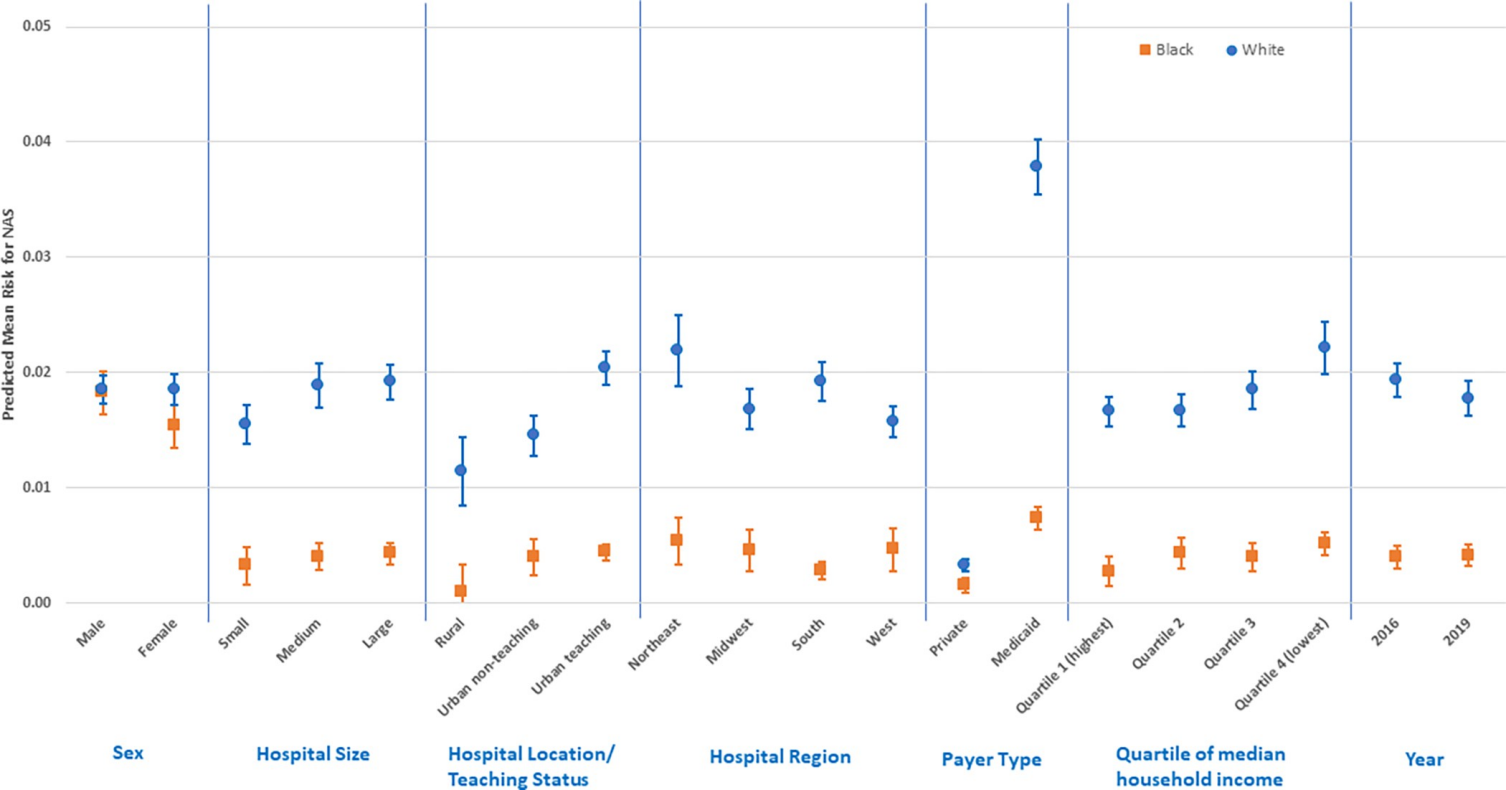
Predictive margins for factors related to the White/Black NAS disparity (risk differences and 95% confidence intervals).

**Fig 2:**
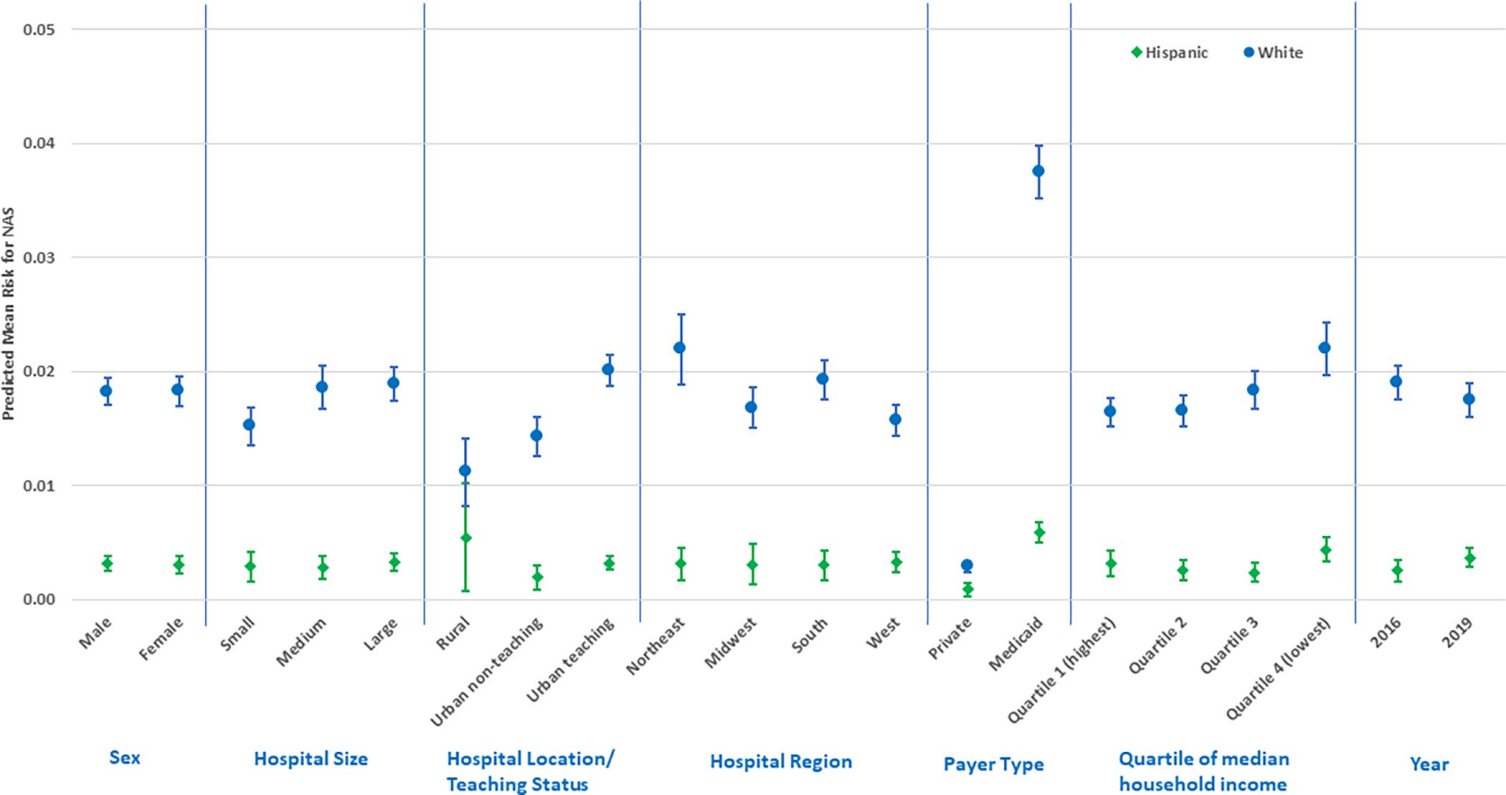
Predictive margins for factors related to the White/Hispanic NAS disparity (risk differences and 95% confidence intervals).

**Fig 3:**
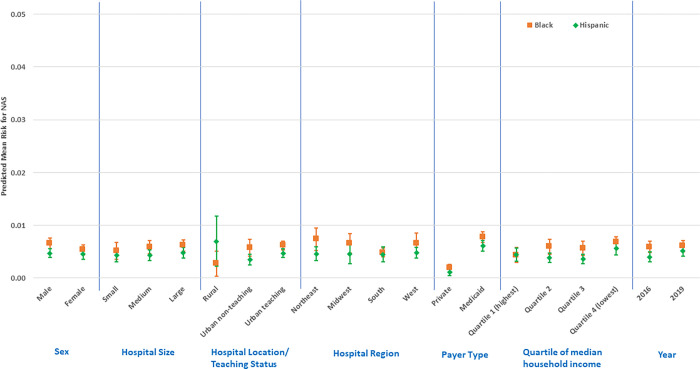
Predictive margins for factors related to Black/Hispanic NAS differences (risk differences and 95% confidence intervals).

In fully specified GLM models (S1–S3 Tables, and Figs 1–3), NAS status among Whites was 1.45% (95% CI: 1.33, 1.57) higher than Blacks and 1.52% (95% CI: 1.39, 1.64) higher than Hispanics; but NAS among Blacks was only 0.14% higher than Hispanics (95% CI: 0.03, 0.24). For comparisons between Whites and Blacks or Hispanics, NAS status was significantly higher for Whites across all factor levels except for rural hospital type, where there was no difference between Whites and Hispanics (all p < 0.05). NAS risk was highest among Whites on Medicaid (RD: 3.79%; 95% CI: 3.55, 4.03) compared to Whites on private insurance (RD: 0.33%; 95% CI: 0.27, 0.38), Blacks with either payer (RD: 0.73%; 95% CI: 0.63, 0.83; RD: 0.15%; 95% CI: 0.08, 0.21), or Hispanics with either payer (RD: 0.59%; 95% CI: 0.50, 0.67; RD: 0.09%; 95% CI: 0.03, 0.15) respectively.

NAS risk among Whites demonstrated a monotonic pattern across ecologic median household income quartiles and was highest in the lowest income quartile (RD: 2.22%; 95% CI: 1.99, 2.44) compared with Blacks (RD: 0.51%; 95% CI: 0.41, 0.61) and Hispanics (RD: 0.44%; 95% CI: 0.33, 0.54) in the same quartile. In addition, compared with Blacks and Hispanics, NAS risk was higher among Whites in medium and large hospitals, urban non-teaching and teaching hospitals; and in the Northeast hospital region (S1 and S2 Tables, and Figs 1 and 2). Joint marginal estimates for Whites with Medicaid plus being in the lowest income quartile further elevated the NAS disparity risk (RD: 4.28%; 95% CI: 3.89, 4.67). This joint estimate was also marginally higher for Whites, with Medicaid, in the lowest income quartile, and hospital status as the Northeast region and urban-teaching (RD: 4.68%; 95% CI: 4.21, 5.14). Comparison of Black to Hispanic NAS risk (S3 Table, and Fig 3) did not reveal any significant difference in stratified estimates.

## Discussion

Our results demonstrate that overall NAS prevalence was 9.84/1000 births and was similar for 2016 and 2019 KID data cycles (i.e., 10.22 and 9.48/1000 births). It should be noted that prior studies using HCUP data and similar ICD-10-CM diagnostic code (i.e., P96.1) have reported overall 2016 prevalence rates of between 6.7 and 7.0/1000 births which is lower than the prevalence observed in the present study [9,12]. We also show that the risk of NAS by race/ethnicity for the combined 2016 and 2019 cycles was 14.72 per/1000 births for Whites, 6.02/1000 for Blacks, and 4.3/1000 for Hispanics, and these were all higher than similar rates reported by AHRQ in 2015 [15]. These differences are likely due to sample restriction to ≥ 35 weeks GA in our study, as this is the subset with higher rates of non-iatrogenic NAS and less likely to be NICU admissions at birth, compared to newborns < 35 weeks GA. To our knowledge, our study is the first to report on NAS race/ethnicity disparities using nationally representative data from the latest 2019 KID data cycle. Although Blacks and Hispanics overall were more likely to be of lower ecologic SES and have Medicaid insurance payer type, it should be noted that low SES and Medicaid payer type, were significantly associated with higher prevalence of NAS in Whites. Although NAS prevalence was similar with regards to hospital designation and regional location in Blacks and Hispanics, we found that hospitals with urban-teaching designation or regional location in the Northeast, were significantly associated with higher prevalence of NAS in Whites (Figs 1 and 2). Further, the largest NAS disparity was observed in Whites with Medicaid in the lowest income quartile, treated in urban-teaching hospitals in the Northeast region (RD: 4.68%; 95% CI: 4.21, 5.14).

Our findings are largely in agreement with several studies that report an increased prevalence of NAS throughout the U.S. in the past two decades [6–9]. However, few of these earlier studies specifically address the race/ethnicity disparity in the prevalence of NAS. Utilizing the KID database for 2000 through 2009 cycles, Patrick et al. reported a substantial increase in the prevalence of NAS (i.e., 1.2 per 1000 births in 2000 increased to 3.39 per 1000 in 2009) and maternal opioid use in the U.S., as well as hospital charges related to NAS [6]. In a subsequent study, the same authors utilized diagnostic and demographic data for hospital discharges from 2009 to 2012 from the KID database and the Nationwide Inpatient Sample (NIS) and showed that NAS prevalence had increased to 5.8 per 1000 hospital births, and that aggregate hospital charges for NAS increased to $1.5 billion, with 81% attributed to state Medicaid programs [7]. Fingar et al. also reported on select HCUP data and showed that between 2006 and 2012, the rate of neonatal hospital stays related to substance use increased by 71%, from 5.1 to 8.7 per 1000 neonatal stays, and associated aggregate hospital costs increased by 135%, from $253 to $595 million [14].

In a similar time period, Villapiano et al. reported on NAS data from 2004 to 2013 and showed that the incidence of NAS and maternal opioid use in the U.S. increased disproportionately in rural counties relative to urban counties [8]. Brown et al. similarly reported that the rate of NAS in a largely rural state, Kentucky, was more than twice that of the national rate [28]. Another study from Nevada using state hospital inpatient administrative data suggests higher prevalence of NAS in Whites, those with Medicaid payer type, and urban rather than rural hospital location [16]. Our findings focus on the NAS race/ethnicity disparity in a national inpatient database and shows that the predominance in Whites occurs largely in hospitals in the Northeast with urban-teaching rather than rural designation.

It has been demonstrated that the U.S. has experienced a rapid increase in NAS as a result of in- utero opioid exposure due to a combination of prescription and recreational opioid use [4,28–30]. Ailes et al. in a CDC report from 2008–2012 showed widespread prescription use in the U.S. among women enrolled in Medicaid compared to those with private insurance [3]. While some mitigation of prescription opioid use has since been achieved by implementation of state and federal policies regulating physician prescribing practices, potential benefits have been attenuated by an increase in recreational drug use among women of reproductive age [28]. The effect of these policies on current NAS trends in the U.S. remain largely unknown, but evidence seem to indicate that the rate of NAS has not slowed, consistent with our study that shows prevalence of NAS has remained relatively unchanged over 2016 and 2019 cycles. Further, a federal law (the “Protect our Infants Act” 2015) was introduced in response to the NAS epidemic, with an aim to combat the demand side of opioid use disorder; however, while it highlights the problem nationally, a major criticism it that the law fails to offer solutions to the rapidly growing problem [28,31].

Corr and Hollenbeak, using KID 2003 through 2012 data, reported that U.S. NAS admissions increased more than fourfold with increased length of stay and associated enormous economic burden, and suggested that NAS was primarily a syndrome of the White, Medicaid-dependent population [13]. More recent studies on NAS using the 2016 KID database have also observed the White-predominance race/ethnicity disparity [9,11,12,32], with Strahan et al. reporting that NAS prevalence had increased to 6.7 per 1000 births, being most common among Whites (10.6 per 1000 births) [12]. Although our results largely agree with these reports, an important limitation of these earlier studies is that many do not specifically detail how iatrogenic cases, that may be mistaken for population-based NAS, were excluded.

While drivers of this disparity remain unknown, one can speculate that both prescription and recreational opioid use contribute to the prevalence of NAS. A 2015 CDC report on opioid prescription claims among women of reproductive age in the U.S. for the period 2008–2012 showed that White women on Medicaid had the highest claims (46.4% vs. 35.2% and 33.6% for Black and Hispanics, respectively) [3]. Interestingly, a 2019 CDC study using self-reported data on prescription opioid use during pregnancy from the Pregnancy Risk Assessment Monitoring System (PRAMS) survey in 32 jurisdictions found that overall 6.6% of women reported opioid use and suggests that use was more frequent in Blacks and Hispanics than Whites (8.6% and 7% vs. 5.9%, respectively), and those with Medicaid insurance (8.5%) [33]. However, this study did not specifically report stratified estimates for Whites on Medicaid. It should be noted that racial disparity in pain management is a well-recognized phenomenon in U.S. healthcare and Hoffman et al. report that Black Americans are systematically undertreated for pain relative to White Americans [34–36], and it is possible that this phenomenon might contribute to the observed White disparity in our study.

Our study has some notable limitations. It should be specified that the unit of analysis was hospital discharges rather than individual newborns. Although a newborn may have more than one admission for the same illness episode, introducing a potential source of bias [37], we consider that the likelihood of duplicate admissions is infrequent in our data analysis and we specifically found no duplicates within each KID cycle. Additionally, the discharge data are obtained from hospital discharge abstracts and these data are subject to misclassification errors and rely on accurate coding. We acknowledge that inconsistencies in the way NAS is defined have been previously reported, and that we were unable to investigate the extent or effect of this issue in the KID database [38]. Further, the KID data cycles are cross-sectional in design and cycle combination assumes that secular data trends are minimal. Our study strengths include use of the largest national population-based database representative of pediatric discharges throughout a diverse range of neonatal units, including NICUs and Normal Newborn Units, in the U.S. In addition, the study years examined included only ICD-10-CM codes, which reduces diagnosis speculation that may afflict earlier studies that include ICD-9 codes, and comprehensively included all diagnostic fields of the discharge records in the KID database. We also specifically excluded iatrogenic cases through use of a dedicated ICD-10-CM code. It should be noted however that a recent CDC study showed that using exposure (P04.49) in addition to withdrawal (P96.1) codes might be more sensitive for identifying NAS than using P96.1 alone (i.e., approximately 12% better that P96.1 only) because P04.49 might identify newborns exhibiting signs of NAS who have not received a diagnosis of withdrawal (P96.1) by providers; however, the authors also acknowledge that a code with higher positive predictive value (PPV) will better identify newborns who genuinely have NAS [39]. As recent increases in NAS are primarily from in utero exposure to opioids [39], for our analysis, we chose to use the code strategy with the highest PPV for identifying newborns who genuinely have NAS, that is P96.1 only (PPV: ≥ 91.7%) [as compared with P04.49 and P96.1 (PPV: 63.9% - 65%)], and we acknowledge that additional studies will be needed to further explore this issue but will likely result in even higher NAS estimates.

Provisional data from CDC’s National Center for Health Statistics indicate that there were an estimated 100,306 drug overdose deaths in the United States during the 12-month period ending in April 2021 [40]. This represents a 28.5% increase from the 78,056 deaths during the same period the previous year [40]. Based on this data, we speculate that there would likely be an increase in NAS prevalence during the COVID-19 pandemic and as such, it would be important to examine the evolution of NAS related sociodemographic patterns in the next HCUP-KID cycle.

## Conclusions

Although low ecologic SES and Medicaid payer type were more prevalent overall among Blacks and Hispanics in our study, we found that the highest prevalence of NAS was observed among Whites on Medicaid and in the lowest income quartile (i.e., with low SES). Further, stratified results largely produced NAS joint-effect estimates that were consistently higher for Whites compared with Blacks and Hispanics across several factors (except for rural hospital type, where there was no difference between Whites and Hispanics), and this warrants further investigation.

## Supporting information

S1 Table Predictive margins for factors related to the White/Black NAS disparity. (XLSX)

S2 Table Predictive margins for factors related to the White/Hispanic NAS disparity. (XLSX)

S3 Table Predictive margins for factors related to the Black/Hispanic NAS disparity. (XLSX)
