# Correction: Disparity in neonatal abstinence syndrome by race/ethnicity, socioeconomic status, and geography, in neonates ≥ 35 weeks gestational age

**DOI:** 10.1371/journal.pone.0322318

**Published:** 2025-04-04

**Authors:** Keith A. Dookeran, Marina G. Feffer, Kyla M. Quigley, Phoebe E. Troeller, Chariya A. Christmon, Janine Y. Khan

The fourth author’s name is spelled incorrectly. The correct name is: Phoebe E. Troeller. The correct citation is: Dookeran KA, Feffer MG, Quigley KM, Troeller PE, Christmon CA, Khan JY (2023) Disparity in neonatal abstinence syndrome by race/ethnicity, socioeconomic status, and geography, in neonates ≥ 35 weeks gestational age. PLoS ONE 18(4): e0284040. https://doi.org/10.1371/journal.pone.0284040.

Fig 1 and S1 Table are incorrect. There are errors in the display of sex differences in White/Black NAS disparity. Please see the correct Fig 1 and S1 Table here.

**Fig 1:**
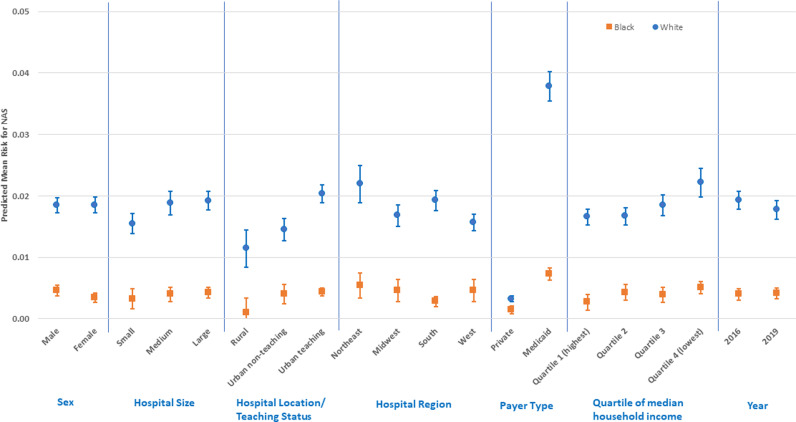
Predictive margins for factors related to the White/Black NAS disparity (risk differences and 95% confidence intervals).

## Supporting information

S1 Table Predictive margins for factors related to the White/Black NAS disparity. (XLSX)
